# Effects of combined basic periodontal treatment and photodynamic therapy on subgingival plaque and inflammatory factors in patients with severe periodontitis

**DOI:** 10.3389/fmed.2026.1933856

**Published:** 2026-10-13

**Authors:** Xue Chen, Yujia Gu, Mei Tian, Huixin Yang, Ge Hu, Qifeng Geng

**Affiliations:** Department of the Fifth Outpatient, Nanjing Stomatological Hospital, Affiliated Hospital of Medical School, Institute of Stomatology, Nanjing University, Nanjing, China

**Keywords:** basic periodontal treatment, clinical efficacy, inflammatory factors, photodynamic therapy, severe periodontitis, subgingival plaque

## Abstract

**Objective:**

This study aimed to investigate the effects of basic periodontal treatment combined with photodynamic therapy on subgingival plaque and inflammatory factors in patients diagnosed with severe periodontitis.

**Methods:**

A retrospective study was conducted on 116 patients with severe periodontitis who were admitted to our hospital between January 2023 and January 2025. Participants were divided into Group A (*n* = 58, periodontal basic treatment) and Group B (*n* = 58, periodontal basic treatment + photodynamic therapy) based on the treatment modality. Clinical efficacy, periodontal status before treatment and 6 months post-treatment, subgingival plaque, levels of inflammatory factors in gingival crevicular fluid, bone metabolism indicators, oral-related quality of life, and incidence of complications were compared between the two groups.

**Results:**

The total effective rate of treatment in group B was higher than that in group A (*P* < 0.05). After 6 months of treatment, the PD, AL, SBI, and PLI levels in group B were lower than those in group A (*P* < 0.05). The total bacterial load of Aggregatibacter actinomycetemcomitans (Aa), *Porphyromonas gingivalis* (Pg), *Prevotella intermedia* (Pi), and plaque in Group B was lower than that in Group A (*P* < 0.05). The levels of interleukin-6 (IL-6), interleukin-1β (IL-1β), tumor necrosis factor-α (TNF-α), and C-reactive protein (CRP) in the gingival crevicular fluid of group B were lower than those of group A (*P* < 0.05). The levels of alkaline phosphatase (ALP), C-terminal peptide of type I collagen (CTX), and osteoclast differentiation factor (RANKL) in group B were lower than those in group A, whereas the level of osteoprotegerin (OPG) was higher than that in group A (*P* < 0.05). The Oral Health Impact Scale-14 (OHIP-14) score in group B was lower than in group A (*P* < 0.05). No statistically significant difference was observed in the incidence of complications between the two groups (*P* > 0.05).

**Conclusion:**

The combination of basic periodontal treatment and photodynamic therapy is effective in treating severe periodontitis. It can effectively improve patients’ periodontal conditions, reduce subgingival plaque and gingival inflammation, improve oral bone metabolism, enhance oral-related quality of life, and is well tolerated.

## 1 Introduction

Periodontitis is a chronic inflammatory disease of gums caused by subgingival plaque. It is a significant cause of tooth loss among adults (1). Severe periodontitis, the final stage of disease progression, is characterized by severe destruction of periodontal supporting tissues, formation of deep periodontal pockets, and recurrent acute symptoms, which seriously affect the oral functions of patients. Chronic inflammation is also closely related to various systemic diseases (2). Currently, clinical treatment for patients with severe periodontitis, mostly involves basic periodontal therapy. Through mechanical means, subgingival plaque biofilm and tartar are removed, which can alleviate gingival inflammation, reduce periodontal probing depth, and delay disease progression (3).

However, accumulating evidence indicates that conventional mechanical instruments fail to access complex anatomical niches at the pocket base and root surface, frequently leaving residual pathogenic bacteria behind. Residual plaque biofilms can continuously release endotoxins and toxic metabolites, stimulate local inflammatory reactions, and progressively damage periodontal tissue, resulting in some patients not achieving ideal therapeutic effects after basic treatment, which has obvious limitations (4). Photodynamic therapy is non-invasive antibacterial auxiliary technology, that combines specific wavelength lasers with photosensitizers. The photosensitizers are selectively enriched in the targeted pathogenic bacteria, and under laser irradiation, reactive oxygen species are produced to rapidly kill the pathogenic bacteria through oxidation. It has shown good therapeutic effects in the treatment of various oral diseases (5, 6). Although some studies have preliminarily explored the clinical efficacy of photodynamic therapy in treating periodontitis, its effects on subgingival plaque and inflammatory factors in patients with severe periodontitis remain unclear (7). This study aimed to retrospectively evaluate the effect of initial periodontal therapy combined with photodynamic therapy on the levels of inflammatory factors in subgingival plaque and gingival crevicular fluid in patients with severe periodontitis using a propensity score-matched cohort design and to provide evidence-based evidence for optimizing adjuvant treatment.

## 2 Materials and Methods

### 2.1 General information

Clinical data were retrospectively collected from patients with severe periodontitis who were admitted to our hospital between January 2023 and January 2025. Preliminary screening identified 186 patients, all of whom had a confirmed diagnosis of severe periodontitis. The inclusion criteria were as follows: met the diagnostic criteria for severe periodontitis (8); age ≥18 years; presence of ≥20 natural teeth; receiving periodontal treatment for the first time; being alert and able to cooperate with all outcome assessments; and voluntary participation in the study, as evidenced by a signed informed consent. The following patients were excluded from the study: those suffering from severe systemic diseases such as cardiovascular or cerebrovascular disease, diabetes, autoimmune diseases, or coagulation disorders; patients who smoked ≥10 cigarettes per day and were unable to quit during the study period; those who had used antibiotics, topical periodontal medications, or anti-inflammatory drugs within the past 6 months; pregnant or lactating women; those with contraindications to photosensitizing agents or laser therapy; those on long-term anticoagulant or antiplatelet therapy; those with aggressive periodontitis or periodontitis associated with systemic diseases; and those with a history of fixed orthodontic treatment. This study was formally approved by the Ethics Committee of the Nanjing Stomatological Hospital, Affiliated Hospital of Medical School, Institute of Stomatology, Nanjing University.

### 2.2 Sample formation: propensity score matching

Among the 186 patients initially screened, 98 received basic periodontal treatment combined with photodynamic therapy at our hospital (Group B), and 88 received basic periodontal treatment (Group A). To minimize selection bias and ensure balanced and comparable baseline data between groups, propensity score matching (PSM) was used for 1:1 pairing. The propensity score calculation was based on a logistic regression model incorporating baseline covariates that may affect treatment efficacy and disease prognosis, including age, gender, body mass index (BMI), periodontitis stage, number of teeth, baseline periodontal probing depth (PD), and baseline gingival crevicular inflammatory factor (IL-6) levels. The propensity score of each patient was calculated based on whether they received photodynamic combined therapy as the dependent variable. Using the nearest-neighbor matching method, the deviation width was set to 0.02, and the matching process strictly followed the principle of “no replacement matching.” Seventy patients were excluded from the final matched cohort due to the absence of eligible matching partners within the predefined 0.02 caliper or missing key covariate data points. A STROBE-compliant patient flow diagram (Figure 1) is provided to illustrate the complete screening, exclusion, and matching process. After PSM processing, 58 pairs of highly matched paired samples (116 patients) were generated and included in the final analysis. All matched covariates had a standardized mean square error of less than 0.1 after matching, indicating a good balance between the two groups.

**Figure 1:**
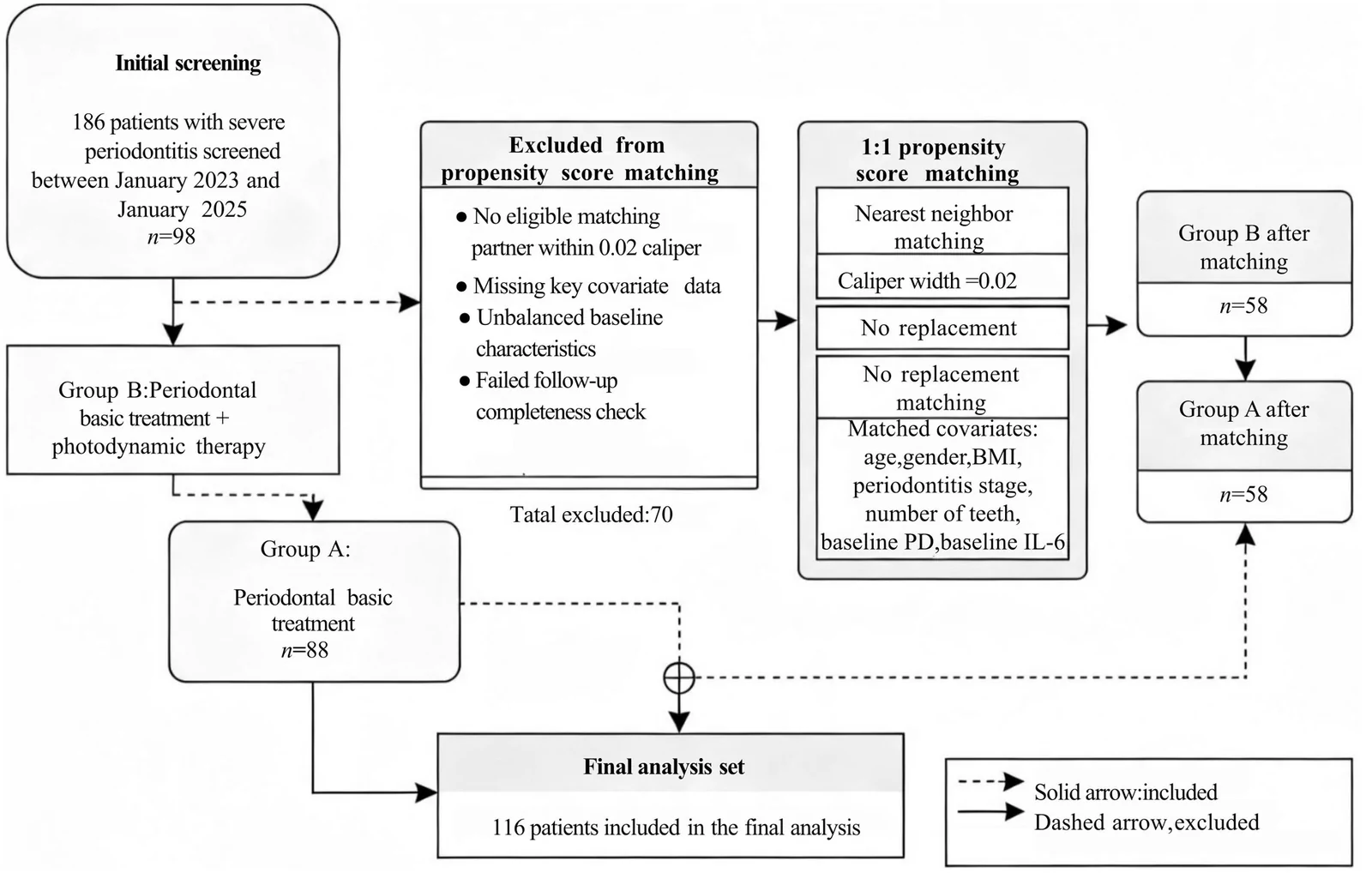
STROBE-compliant patient flow diagram.

### 2.3 Methods

Both groups received oral hygiene instructions prior to treatment, including guidance on proper tooth brushing, flossing, and interdental brush techniques, to ensure patient compliance with subsequent treatment and postoperative maintenance. All treatment procedures were performed by a senior periodontist.

Group A received basic periodontal treatment. An ultrasonic scaler was used to remove supragingival plaque, soft deposits, and calculus from each tooth surface. A periodontal probe was used to measure the periodontal pocket depth and determine the distribution of calculus and plaque. Local anesthesia was administered using a 2% lidocaine injection (Beijing Yimin Pharmaceutical, National Drug Approval No. H11020322, Specification: 2 mL:40 mg) for local anesthesia. Anesthesia sites were selected based on the extent of the patient's periodontal lesions, and the solution was slowly injected subgingivally around the periodontal pockets, with 0.5–1.0 mL per injection site. It is imperative to wait 5–10 min following the injection to ascertain that the patient's local sensation has diminished and there is no significant pain before proceeding with subgingival scaling and root planing to remove subgingival bacterial biofilm, calculus, and contaminated dentin. Scaling should be performed gently to avoid damaging the periodontal attachment epithelium and ensure that the root surface is smooth and flat. After the procedure, the periodontal pocket was rinsed with saline to remove residual debris. Patients were instructed to avoid spicy, hard, and extremely hot or cold foods for one week post-procedure, brush teeth properly twice daily, and attend regular follow-up visits to monitor oral recovery.

Group B received photodynamic therapy in addition to the procedures performed in Group A. Prior to treatment, the periodontal pockets were irrigated with a 3% hydrogen peroxide solution (Guangdong Nanguo Pharmaceutical, concentration: 3%) to remove residual debris and secretions. The use of 3% hydrogen peroxide is a standard preparatory step for PDT; it mechanically clears intrapocket debris and secretions and mildly oxidizes the pocket environment to improve subsequent photosensitizer penetration and antimicrobial efficacy. This irrigation procedure was not considered an independent therapeutic intervention, and all patients in Group A received an equivalent volume of saline irrigation as a control. After drying, a 0.1% methylene blue solution (Jiangsu Yongtai Pharmaceutical Co. Ltd. China) was used as the photosensitizer, with approximately 0.1 mL delivered into each periodontal pocket via a fine irrigation needle to fully cover the inner pocket walls and root surfaces. The area was kept away from light for 20 min to ensure that the photosensitizer was fully absorbed by the pathogenic bacteria within the periodontal pockets (photosensitizer-to-light interval: 20 min). A semiconductor laser therapy unit (Hainan Sandun Medical, Model: SUNDOM-300IB) was set to a wavelength of 635 nm, output power of 80 mW, and spot diameter of 0.8 mm. The laser fiber was slowly inserted along the periodontal pocket wall to the bottom of the pocket. Each periodontal pocket was irradiated using continuous irradiation, and the irradiation time was adjusted based on the depth of the periodontal pocket, with 1 min of irradiation per millimeter of pocket depth with a maximum irradiation time of 5 min per periodontal pocket. During the procedure, the laser beam should not be directed toward the gingival mucosa or normal tissues. The tip of the laser fiber was moved at a constant speed to prevent overheating and tissue damage. Photodynamic therapy was performed once a week for three consecutive sessions.

### 2.4 Observation criteria

(1) Clinical Efficacy. Reference (8) was used for the evaluation 6 months post-treatment. This composite efficacy endpoint was defined based on established periodontal therapeutic evaluation criteria, incorporating both clinical symptom improvement and objective periodontal parameter changes, and has been widely adopted in previous studies. Marked improvement: Significant improvement in clinical symptoms, such as gingival bleeding, swelling, and inflammation; periodontal pocket depth (PD) reduced by ≥2 mm compared to pre-treatment; and gingival index (GI) decreased by >40%. Improvement: Some improvement in the aforementioned clinical symptoms; PD reduced by 1 mm compared to pre-treatment; and GI decreased by 20%–40%. No improvement: did not meet the above criteria. Overall response rate = (Marked improvement + Improvement)/Total number of cases × 100%.
(2) Periodontal status. All periodontal examinations were performed by a single calibrated senior periodontist using a Williams periodontal probe with a standardized probing force of 20–25 g. Six sites per tooth (mesiobuccal, midbuccal, distobuccal, mesiolingual, midlingual, and distolingual) were examined for each selected target tooth. Intra-examiner reproducibility was validated prior to study initiation via duplicate measurements in 10% of the enrolled patients, yielding an intraclass correlation coefficient (ICC) > 0.90 for both PD and AL. All outcome assessments were conducted by an examiner who was blinded to the patient's treatment allocation. A single periodontist used standardised periodontal probes to assess parameters such as PD, clinical attachment loss (AL), sulcus bleeding index (SBI), and plaque index (PLI) at baseline and six-month follow-up. SBI criteria (9): 0 = No bleeding upon probing; 1 = Punctate bleeding at the gingival margin upon probing; 2 = Bleeding spreading along the gingival margin upon probing; 3 = Significant bleeding extending beyond the gingival margin upon probing; and 4 = Spontaneous gingival bleeding. PLI grading criteria (10): 0 = No plaque on the tooth surface; 1 = Thin plaque at the gingival margin, not easily visible to the naked eye and detectable only with a probe; 2 = Visible plaque at the gingival margin and on adjacent surfaces; and 3 = Extensive plaque covering the gingival margin and part of the tooth surface.
(3) Subgingival plaque. Subgingival plaque samples were collected from patients who had fasted for 8 h at baseline and again 6 months after treatment. The same three teeth with the deepest periodontal pockets and the most pronounced inflammation identified at baseline were sampled at both time points to ensure longitudinal consistency. Plaque was gently scraped from 1 to 3 mm below the gingival margin using a sterile periodontal probe and placed into a centrifuge tube containing 1 mL of sterile saline. The tubes were vortexed to obtain a uniform plaque suspension. For total bacterial load quantification, serial dilutions of the plaque suspension were inoculated onto blood agar plates and incubated at 37 °C in a 5% CO_2_ incubator for 48 h. The total bacterial load was expressed as colony-forming units per milliliter (CFU/mL). For the quantitative analysis of specific periodontal pathogens, bacterial DNA was extracted from the plaque suspension using the TIANamp Bacteria DNA Kit (Tiangen Biotech Co. Ltd. Beijing, China, Cat. No. DP302) according to the manufacturer's instructions. Quantitative polymerase chain reaction (qPCR) was performed using the TB Green Premix Ex Taq II kit (Takara Bio Inc., Shiga, Japan; Cat. No. RR820A) on a LightCycler 480 II real-time PCR system (Roche Applied Science, Mannheim, Germany). The universal 16S rRNA gene was used as an internal reference for normalization. The primer sequences were as follows: Aggregatibacter actinomycetemcomitans (Aa) forward 5'-GCC TAC CTA TGG TGG AAG ACG-3', reverse 5'-CCC ACT CTA CGA GCT CCA GAC-3' (amplicon 220 bp); Porphyromonas gingivalis (Pg) forward 5'-AGG CAG CTT GCC ATA CTG CG-3', reverse 5'-ACT GTT AGC AAC TAC CGA TGT-3' (amplicon 175 bp); Prevotella intermedia (Pi) forward 5'-TTG TTG GGG AAT GGG ATG TTC-3', reverse 5'-ACA AAG CGG TAT TTA CCA GCA-3' (amplicon 180 bp); and 16S rRNA forward 5'-CCT ACG GGA GGC AGC AG-3', reverse 5'-GGA CTA CCA GGG TAT CTA ATC CTG TT-3' (amplicon 466 bp). All primer sequences were verified against the GenBank database and confirmed to specifically match the target species. The thermal cycling conditions comprised initial denaturation at 95 °C for 30 s, followed by 40 cycles at 95 °C for 5 s and 60 °C for 30 s. Melting curve analysis was performed to confirm the amplification specificity. The relative abundance of each target bacterium was normalized to the 16S rRNA gene and calculated using the 2^−ΔΔCt^ method. Negative controls (no template) and positive controls (known reference strains) were included in each qPCR run.
(4) Inflammatory factor levels in the gingival crevicular fluid. Gingival crevicular fluid samples were collected from patients in a fasting state both before and 6 months after treatment. Prior to collection, the tooth surface at the collection site was cleaned with sterile saline and the surface moisture was blotted dry. A sterile filter paper strip measuring 1 mm in width and 8 mm in length was inserted into the target periodontal pocket, left in place for 30 s, and then removed to ensure that the strip was fully saturated and free of blood contamination. The wet weight of the filter paper strip was weighed using an electronic balance, and the dry weight was subtracted to calculate the volume of the gingival crevicular fluid. The filter paper strip was placed into a centrifuge tube containing 200 μL of sterile phosphate-buffered saline (PBS), shaken at 4 °C for 30 min, and centrifuged at 3,000 rpm for 10 min. The supernatant was collected and stored at −80 °C for later analysis. The levels of IL-6, interleukin-1β (IL-1β), tumor necrosis factor-α (TNF-α), and C-reactive protein (CRP) in gingival crevicular fluid were measured using the enzyme-linked immunosorbent assay (ELISA) method. The procedures were strictly followed according to the instructions.
(5) Bone metabolism marker. Using the gingival crevicular fluid samples described above, the levels of alkaline phosphatase (ALP), C-terminal telopeptide of type I collagen (CTX), osteoprotegerin (OPG), and receptor activator of nuclear factor kappa-B ligand (RANKL) were measured using the ELISA method. The procedures were strictly performed according to the manufacturer's instructions.
(6) Oral health-related quality of life. The Oral Health Impact Profile-14 (OHIP-14) (11) was used to assess patients’ oral-related quality of life both before treatment and 6 months after treatment. This scale comprises seven dimensions: functional limitations, physical pain, psychological distress, physical barriers, psychological barriers, social barriers, and disability. Each dimension contains two items, for a total of 14 items. Each item is scored using a 5-point Likert scale ranging from 0 to 4, with a maximum score of 56. Higher scores indicate a poorer oral health-related quality of life.
(7) Incidence of complications. Complications were recorded and stratified into two periods: the active treatment phase and the 0–6 month post-treatment follow-up phase, to distinguish acute procedural adverse events from delayed post-treatment complications. The occurrence of complications during treatment and within 6 months post-treatment was recorded for both groups, including gingival bleeding, periodontal abscesses, localized mucosal redness and swelling, and infection. The incidence of complications was calculated as follows: incidence of complications = number of cases with complications/total number of cases × 100%.

### 2.5 Statistical methods

The data were entered and analysed using SPSS 27.0. Categorical data are described as [*n*(%)] and were analysed using the chi-square test or Fisher's exact test. For ordinal data, the rank-sum test was performed; for continuous data following a normal distribution, values are described using $\left(\bar{x}\;\pm s\right)$. For between-group comparisons after PSM, the independent-samples *t*-test was used because the matched cohorts were treated as independent samples with balanced baseline covariates. For within-group comparisons between baseline and follow-up, the paired-samples *t*-test was used. For continuous data not following a normal distribution, values were expressed as *M* (*Q*1, *Q*3); and for comparisons between groups, the Mann–Whitney *U* test was used. For all primary between-group comparisons, 95% confidence intervals (CI) and Cohen's *d* effect sizes were calculated to quantify the magnitude of the treatment effects. Statistical significance was set at *p* < 0.05.

## 3 Results

### 3.1 Comparison of general characteristics between the two groups

Comparison of the general characteristics between the two groups revealed no statistically significant differences (*P* > 0.05) (Table 1).

**Table 1:** Comparison of general characteristics between the two groups [*n* (%), $\bar{x}\pm s$, *M* (*Q*1, *Q*3)].

Group	Age (years)	Gender		BMI (kg/m^2^)	Stage		Number of teeth
		Male	Female		III	IV	
Group A (*n* = 58)	43.16 ± 9.02	31 (53.45)	27 (46.55)	22.70 ± 2.26	42 (72.41)	16 (27.59)	27.00 (25.00, 28.00)
Group B (*n* = 58)	44.48 ± 6.87	28 (48.28)	30 (51.72)	22.66 ± 2.07	40 (68.97)	18 (31.03)	27.00 (26.00, 28.00)
*t*/*χ*^2^/*U*	0.892	0.310		0.120	0.166		0.314
*P*	0.374	0.577		0.905	0.683		0.754

### 3.2 Comparison of clinical efficacy between the two groups

The overall response rate in Group B was higher than that in Group A (*P* *<* 0.05) (Table 2).

**Table 2:** Comparison of clinical efficacy between the two groups [*n* (%)].

Group	Marked improvement	Partial response	No effect	Overall response rate
Group A (*n* = 58)	21 (36.21)	22 (37.93)	15 (25.86)	43 (74.14)
Group B (*n* = 58)	36 (62.07)	17 (29.31)	5 (8.62)	53 (91.38)
*χ* ^2^				6.042
*P*				0.014

### 3.3 Comparison of periodontal status between the two groups before treatment and 6 months after treatment

At 6 months post-treatment, Group B had lower levels of PD (MD = 1.00, 95% CI: 0.85–1.15), AL (MD = 0.90, 95% CI: 0.76–1.03), SBI (MD = 0.36, 95% CI: 0.20–0.53), and PLI (MD = 0.14, 95% CI: 0.22–0.25) than Group A (*P* *<* 0.05) (Table 3, Figure 2).

**Table 3:** Comparison of periodontal status between the two groups before treatment and 6 months after treatment $\left(\bar{x}\pm s\right)$.

Group	PD (mm)		AL (mm)		SBI		PLI	
	Before treatment	6 months post-treatment	Before treatment	6 months after treatment	Before treatment	6 months after treatment	Before treatment	6 months after treatment
Group A (*n* = 58)	6.29 ± 0.53	4.16 ± 0.41*	5.62 ± 0.49	3.07 ± 0.37*	2.48 ± 0.50	1.53 ± 0.50*	2.07 ± 0.32	1.05 ± 0.35*
Group B (*n* = 58)	6.24 ± 0.51	3.16 ± 0.41*	5.71 ± 0.53	2.17 ± 0.38*	2.55 ± 0.57	1.17 ± 0.38*	2.10 ± 0.41	0.91 ± 0.28*
*T*	0.537	13.120	0.910	12.887	0.692	4.369	0.510	2.347
*P*	0.592	<0.001	0.365	<0.001	0.490	<0.001	0.611	0.021

Compared with pre-treatment values.

* *P* *<* 0.05.

**Figure 2:**
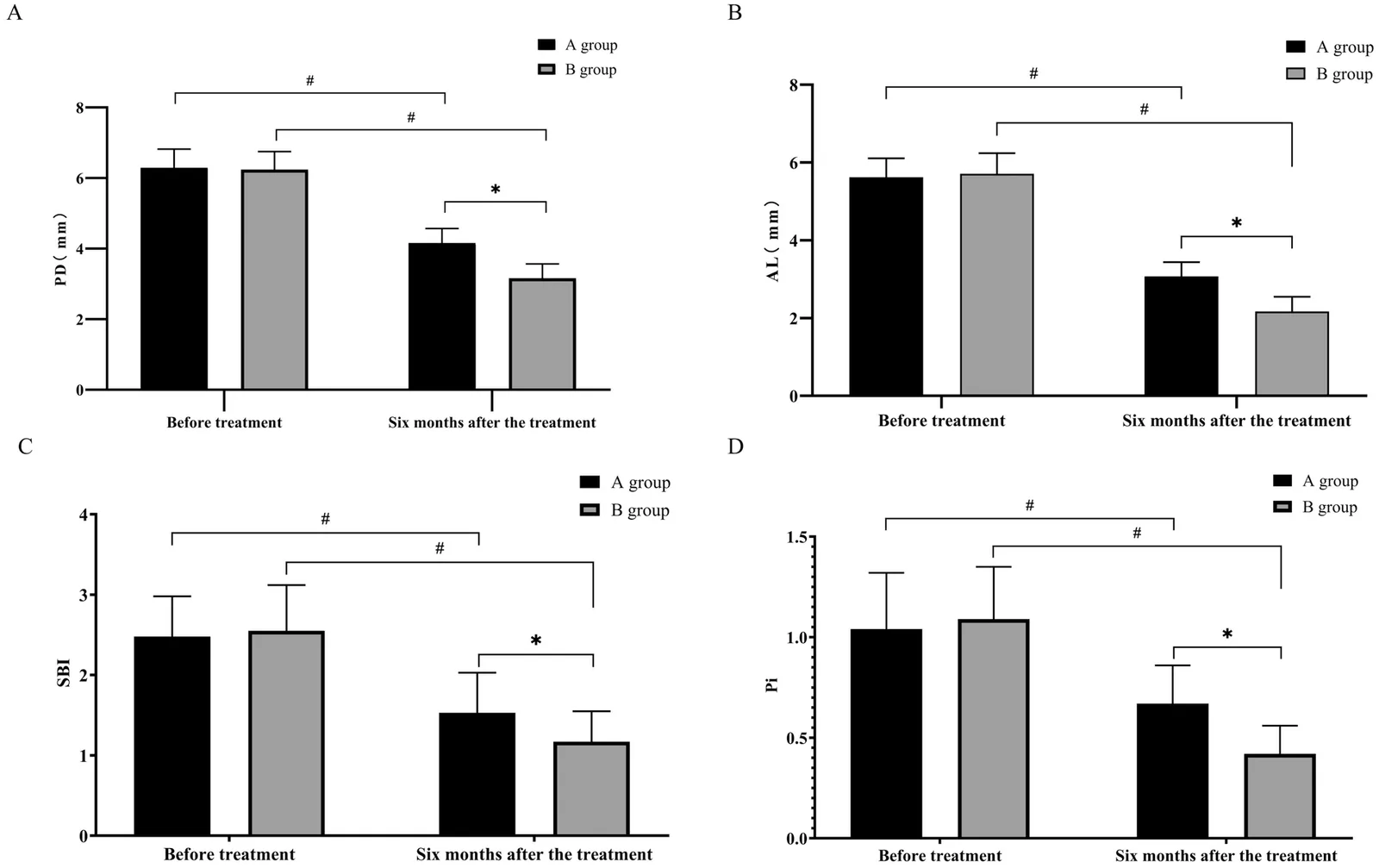
Comparison of periodontal status between the two groups before treatment and 6 months after treatment. **(A)** Comparison of PD between the two groups before treatment and 6 months after treatment; **(B)** comparison of AL between the two groups before treatment and 6 months after treatment; **(C)** comparison of SBI between the two groups before treatment and 6 months after treatment; **(D)** comparison of PLI between the two groups before treatment and 6 months after treatment; compared with Group A, *P < 0.05; compared with the same group before treatment, ^#^*P* < 0.05.

### 3.4 Comparison of subgingival plaque between the two groups before treatment and 6 months after treatment

Six months after treatment, Group B had lower levels of Aa (MD = 0.28, 95% CI: 0.23–0.34), Pg (MD = 0.22, 95% CI: 0.17–0.28), Pi (MD = 0.26, 95% CI: 0.19–0.32), and total bacterial load in subgingival plaque (MD = 1.65, 95% CI: 1.34–1.96) than Group A (*P* *<* 0.05) (Table 4, Figure 3).

**Table 4:** Comparison of subgingival plaque between the two groups before treatment and 6 months after treatment $\left(\bar{x}\pm s\right)$.

Group	Aa		Pg		Pi		Total bacterial load in plaque (×10^5^ CFU/mL)	
	Before treatment	6 months after treatment	Before treatment	6 months post-treatment	Before treatment	6 months after treatment	Before treatment	6 months after treatment
Group A (*n* = 58)	0.99 ± 0.25	0.64 ± 0.16*	1.00 ± 0.23	0.57 ± 0.17*	1.04 ± 0.28	0.67 ± 0.19*	8.69 ± 1.63	4.32 ± 0.86*
Group B (*n* = 58)	1.02 ± 0.33	0.36 ± 0.12*	1.05 ± 0.23	0.35 ± 0.10*	1.09 ± 0.26	0.42 ± 0.14*	9.10 ± 1.52	2.67 ± 0.82*
*T*	0.561	10.796	1.259	8.457	1.059	8.125	1.398	10.573
*P*	0.576	<0.001	0.210	<0.001	0.292	<0.001	0.165	<0.001

Compared with pre-treatment levels.

* *P* *<* 0.05.

**Figure 3:**
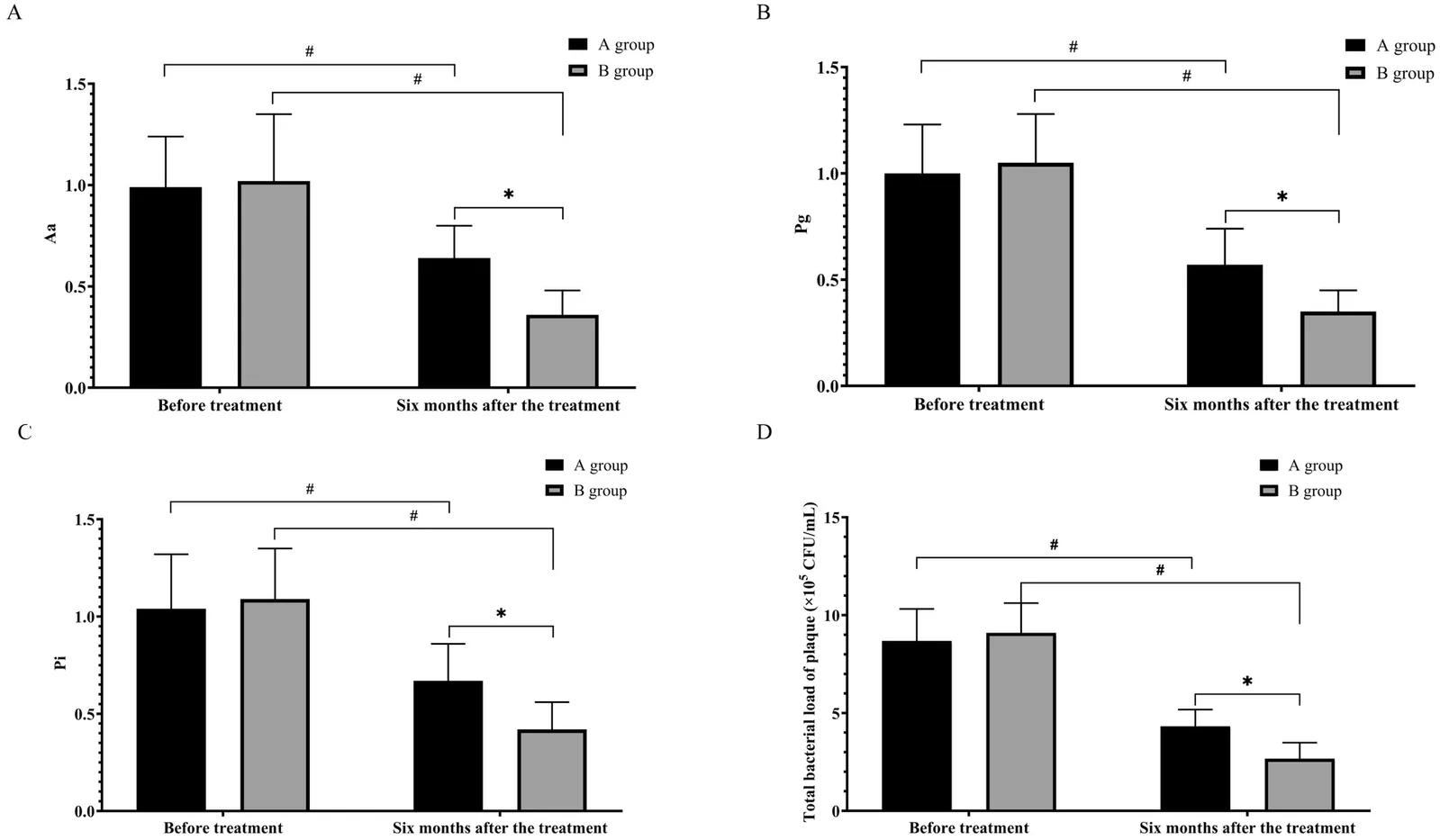
Comparison of subgingival plaque between the two groups before treatment and 6 months after treatment. **(A)** Comparison of Aa levels between the two groups before treatment and 6 months after treatment; **(B)** comparison of Pg levels between the two groups before treatment and 6 months after treatment; **(C)** comparison of Pi levels between the two groups before treatment and 6 months after treatment; **(D)** comparison of total bacterial load in plaque between the two groups before treatment and 6 months after treatment; compared with Group A, *P < 0.05; compared with the same group before treatment, ^#^*P* < 0.05.

### 3.5 Comparison of inflammatory cytokine levels in gingival crevicular fluid between the two groups before treatment and 6 months after treatment

Six months after treatment, the levels of IL-6 (MD = 7.31, 95% CI: 5.87–8.75), IL-1β (MD = 8.34, 95% CI: 6.20–10.47), TNF-α (MD = 5.45, 95% CI: 3.94–6.96), and CRP (MD = 3.11, 95% CI: 2.15–4.08) in Group B were all lower than those in Group A (*P* *<* 0.05) (Table 5, Figure 4).

**Table 5:** Comparison of inflammatory cytokine levels in gingival crevicular fluid between the two groups before treatment and 6 months after treatment $\left(\bar{x}\pm s\right)$.

Group	IL-6 (pg/mL)		IL-1β (pg/mL)		TNF-α (pg/mL)		CRP (pg/mL)	
	Before treatment	6 months post-treatment	Before treatment	6 months post-treatment	Before treatment	6 months after treatment	Before treatment	6 months after treatment
Group A (*n* = 58)	33.00 ± 5.44	18.84 ± 3.42*	45.62 ± 7.52	24.60 ± 5.55*	29.48 ± 5.68	15.63 ± 4.76*	20.37 ± 4.78	10.69 ± 3.04*
Group B (*n* = 58)	33.71 ± 6.01	11.53 ± 4.34*	45.52 ± 7.09	16.27 ± 6.05*	27.77 ± 5.33	10.18 ± 3.31*	20.10 ± 4.53	7.57 ± 2.13*
*T*	0.672	10.075	0.067	7.739	1.672	7.163	0.313	6.378
*P*	0.503	<0.001	0.946	<0.001	0.097	<0.001	0.755	<0.001

Compared with pre-treatment levels.

* *P* *<* 0.05.

**Figure 4:**
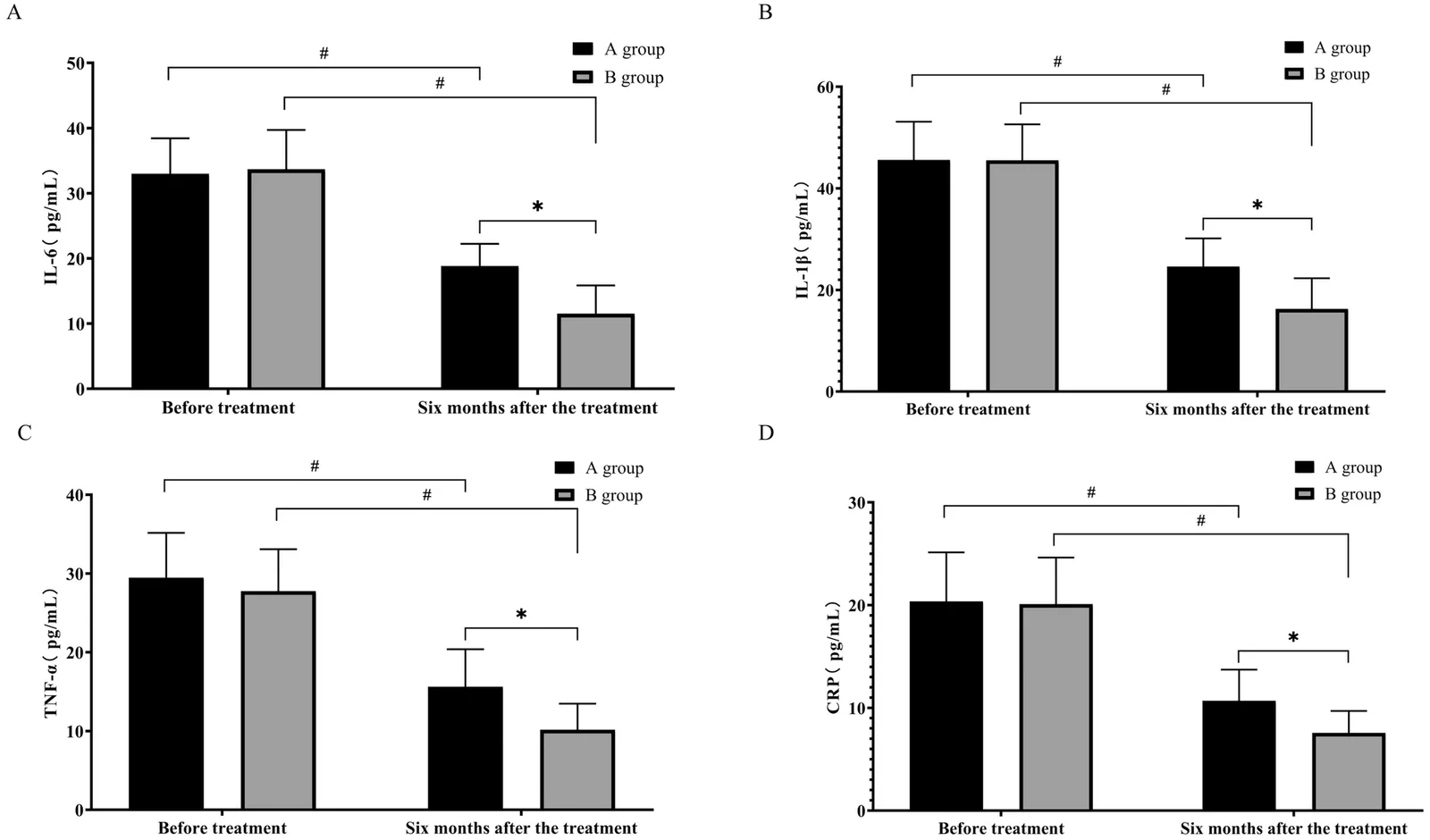
Comparison of inflammatory cytokine levels in gingival crevicular fluid between the two groups before treatment and 6 months after treatment. **(A)** Comparison of IL-6 levels between the two groups before treatment and 6 months after treatment; **(B)** comparison of IL-1β levels between the two groups before treatment and 6 months after treatment; **(C)** comparison of TNF-α levels between the two groups before treatment and 6 months after treatment; **(D)** comparison of CRP levels between the two groups before treatment and 6 months after treatment; compared with Group A, *P < 0.05; compared with pre-treatment levels within the same group, ^#^*P* < 0.05.

### 3.6 Comparison of bone metabolism markers between the two groups before treatment and 6 months after treatment

Six months after treatment, Group B had lower levels of ALP (MD = 16.06, 95% CI: 12.75–19.36), CTX (MD = 1.11, 95% CI: 0.78–1.44), and RANKL (MD = 8.91, 95% CI: 6.52–11.30) than Group A, and higher levels of OPG (MD = −11.74, 95% CI: −15.72 to 7.76) than Group A (*P* *<* 0.05) (Table 6, Figure 5).

**Table 6:** Comparison of bone metabolism markers between the two groups before treatment and 6 months after treatment $\left(\bar{x}\pm s\right)$.

Group	ALP (U/L)		CTX (pg/mL)		OPG (pg/mL)		RANKL (pg/mL)	
	Before treatment	6 months post-treatment	Before treatment	6 months post-treatment	Before treatment	6 months after treatment	Before treatment	6 months after treatment
Group A (*n* = 58)	89.67 ± 13.01	70.27 ± 9.26*	5.87 ± 1.24	3.89 ± 1.07*	41.75 ± 7.98	58.12 ± 10.58*	38.12 ± 6.51	25.46 ± 6.27*
Group B (*n* = 58)	93.63 ± 13.87	54.21 ± 8.70*	5.83 ± 1.38	2.78 ± 0.69*	44.05 ± 9.84	69.86 ± 11.05*	38.69 ± 7.61	16.55 ± 6.72*
*T*	1.586	9.624	0.170	6.633	1.384	5.844	0.435	7.385
*P*	0.115	<0.001	0.865	<0.001	0.169	<0.001	0.664	<0.001

Compared with pre-treatment levels.

* *P* *<* 0.05.

**Figure 5:**
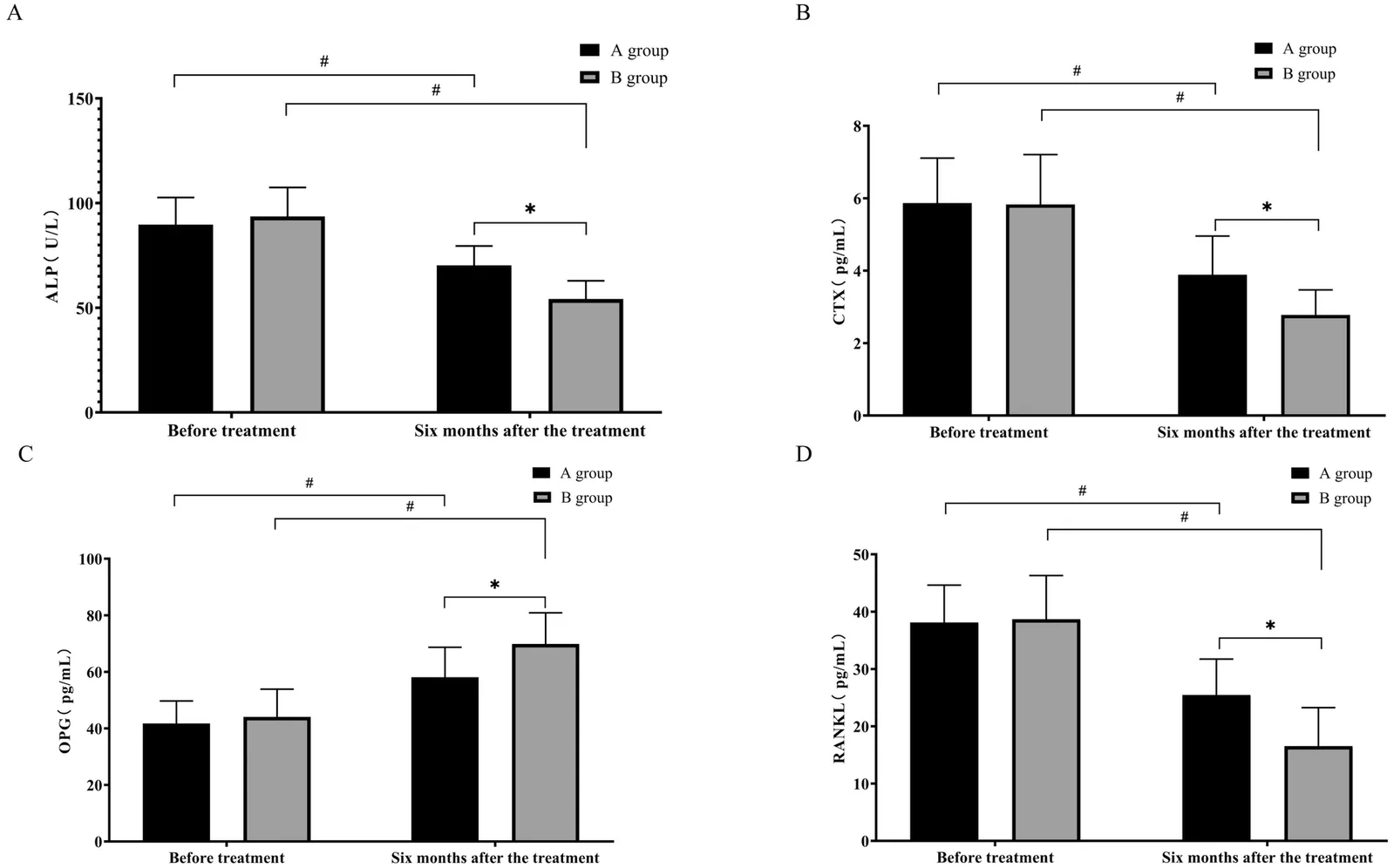
Comparison of bone metabolism markers between the two groups before treatment and 6 months after treatment. **(A)** Comparison of ALP levels between the two groups before treatment and 6 months after treatment; **(B)** comparison of CTX levels between the two groups before treatment and 6 months after treatment; **(C)** comparison of OPG levels between the two groups before treatment and 6 months after treatment; **(D)** comparison of RANKL levels between the two groups before treatment and 6 months after treatment; compared with Group A, *P < 0.05; compared with pre-treatment levels within the same group, ^#^*P* < 0.05.

### 3.7 Comparison of oral health related quality of life between the two groups before treatment and 6 months after treatment

Six months after treatment, Group B had a lower OHIP-14 score than Group A (MD = 6.60, 95% CI: 5.37–7.83, *P* *<* 0.05) (Table 7, Figure 6).

**Table 7:** Comparison of oral health-related quality of life between the two groups before treatment and 6 months after treatment $\left(\bar{x}\pm s\right)$.

Group	OHIP-14 (score)	
	Pre-treatment	6 months post-treatment
Group A (*n* = 58)	32.88 ± 5.04	21.05 ± 3.75*
Group B (*n* = 58)	33.81 ± 3.10	14.45 ± 2.88*
*T*	1.199	10.641
*P*	0.233	<0.001

Compared with pre-treatment levels.

* *P* *<* 0.05.

**Figure 6:**
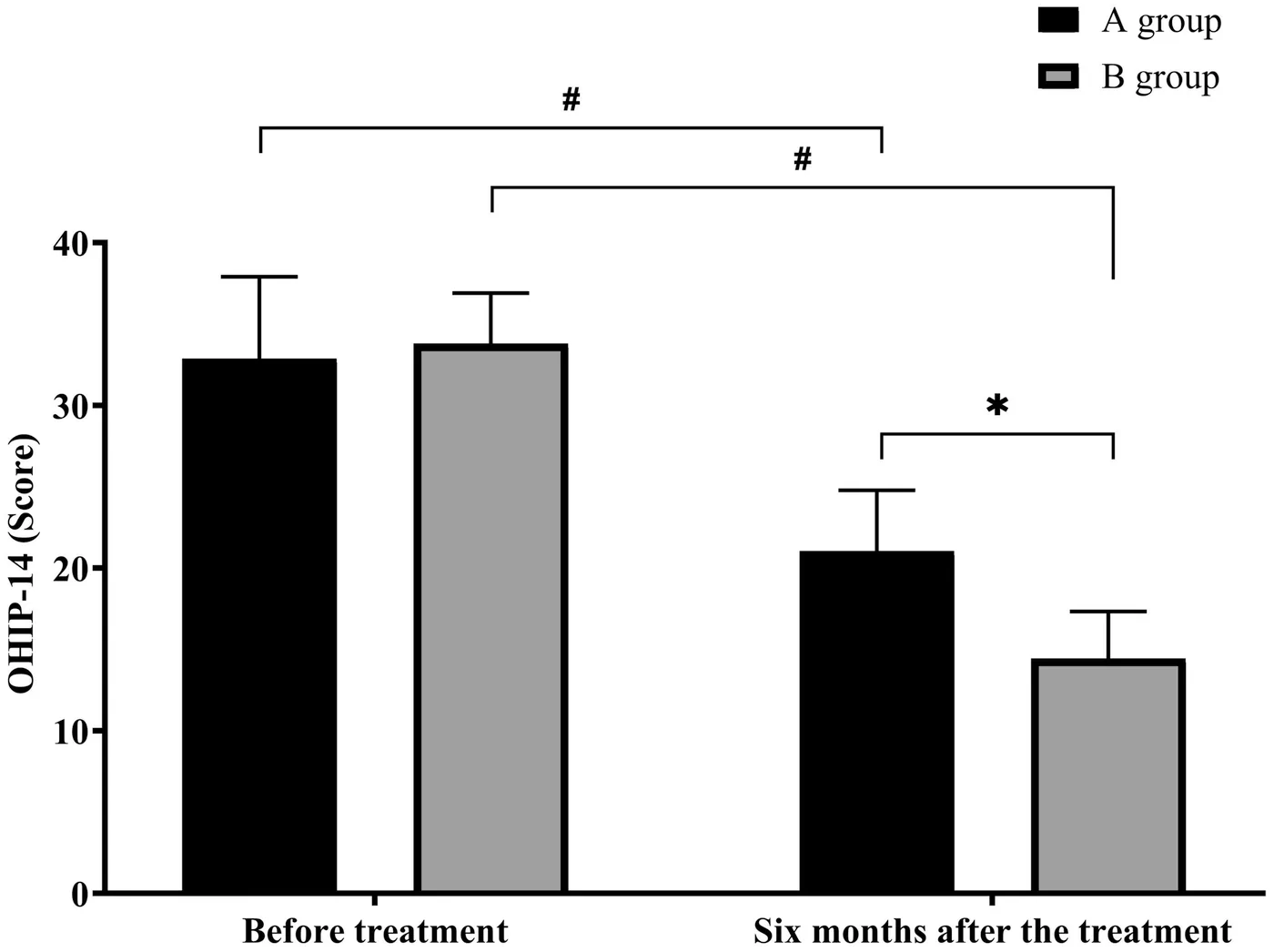
Comparison of oral-related quality of life between the two groups before treatment and 6 months after treatment. Compared with Group A, *P *<* 0.05; compared with pre-treatment values within the same group, ^#^*P* *<* 0.05.

### 3.8 Comparison of the incidence of complications between the two groups

When stratified by time period, no significant intergroup differences were observed in the incidence of acute complications during treatment or delayed complications within 6 months post-treatment (*P* > 0.05). A comparison of the complication rates between the two groups showed no statistically significant difference (*P* > 0.05) (Table 8).

**Table 8:** Comparison of the incidence of complication rates between the two groups [*n* (%)].

Group	During treatment phase			0–6 months post-treatment phase			Overall incidence
	Gingival bleeding	Localized mucosal erythema and edema	Subtotal	Periodontal abscess	Infection	Subtotal	
Group A (*n* = 58)	3 (5.17)	4 (6.90)	7 (12.07)	2 (3.45)	1 (1.72)	3 (5.17)	10 (17.24)
Group B (*n* = 58)	1 (1.72)	2 (3.45)	3 (5.17)	1 (1.72)	0 (0.00)	1 (1.72)	4 (6.90)
*χ* ^2^			1.751			1.036	2.924
*P*			0.186			0.309	0.152

## 4 Discussion

Severe periodontitis is characterized by irreversible destruction of periodontal supporting tissues, manifested by the formation of deep periodontal pockets, significant clinical attachment loss, and alveolar bone resorption exceeding half the root length. It is often accompanied by recurrent gingival redness, swelling, bleeding, periodontal abscesses, and tooth mobility, severely affecting patients’ masticatory function and facial aesthetics (12). Epidemiological surveys indicate that although the prevalence of gingivitis and mild periodontitis has been declining in recent years owing to the widespread availability of oral public health services, the prevalence of severe periodontitis remains high at approximately 10%–15%, with significant individual variation among affected populations (13). The occurrence of severe periodontitis is related to the abnormal colonization of subgingival plaque biofilms and local inflammation in the body. Pathogenic bacteria in subgingival plaque activate host immune cells by releasing toxic products, such as the endotoxins and enzymes, triggering sustained inflammatory reactions and subsequently damaging supporting tissues such as periodontal membrane, dental bone, and alveolar bone, forming a vicious cycle (14). In addition, chronic inflammation associated with severe periodontitis can affect the whole body through blood circulation, interact with diabetes, cardiovascular and cerebrovascular diseases, osteoporosis, and other systemic diseases, and further increase the health burden of patients (15). Currently, the treatment of severe periodontitis is mainly based on the principles of removing the cause, controlling inflammation, and preventing further tissue damage. However, owing to the complexity of the condition and the prolonged course of the disease, a single treatment plan is often difficult to achieve ideal therapeutic effects. Exploring safe and effective combination therapy models has become key to improving patient prognosis and enhancing their quality of life (16).

Aa, Pg, and Pi in subgingival plaque are recognized as core pathogenic bacteria in severe periodontitis; they can destroy periodontal tissues, induce an inflammatory response in the host, and accelerate disease progression (17). PD, AL, SBI, and PLI are common clinical periodontal indices that reflect the extent of destruction of periodontal supporting tissue. Among these, SBI and PLI reflect the status of gingivitis and the effectiveness of plaque control. The results of this study show that the overall treatment efficacy rate in Group B was higher than that in Group A. Six months after treatment, improvements in periodontal indices, Aa, Pg, Pi, and total bacterial load in plaque were all superior in Group B compared to Group A. Conventional periodontal scaling and root planing mechanically remove supragingival and subgingival plaque, calculus, and contaminated dentin, which can inhibit the colonization of the dental biofilm and reduce periodontal inflammation. Photodynamic therapy can act on plaque biofilms, generating reactive oxygen species through the synergistic effect of photosensitizers and specific wavelength lasers, rapidly killing pathogenic bacteria, and not easily developing drug resistance, which can compensate for the limitations of conventional mechanical therapy (18). The combined application of the two schemes can remove most visible plaque and tartar through basic periodontal treatment, creating a favorable treatment environment for photodynamic therapy. Simultaneously, photodynamic therapy can remove residual pathogenic bacteria from mechanical treatment, further inhibit plaque biofilm regeneration, reduce gingival inflammation, lower PD, promote periodontal attachment tissue repair, and improve overall treatment efficiency. Consistent with our findings, a recent systematic review and meta-analysis of indocyanine green-mediated antimicrobial photodynamic therapy adjunctive to scaling and root planing confirmed that adjunctive PDT yields significant additional improvements in PD reduction, CAL gain, and subgingival pathogen clearance compared with SRP alone (19). Another recent review further corroborated the favorable microbiological and clinical outcomes of adjunctive PDT in periodontal populations, noting that the additional benefit was most pronounced in deep periodontal pockets, where mechanical debridement is less effective (20). In the present study, the mean PD reduction of 3.08 mm in the combination group exceeded the 2.13 mm reduction in the SRP-only group, with a between-group difference of 0.95 mm (95% CI: 0.81–1.09), which represents a clinically meaningful improvement, given that a 0.5 mm PD reduction is considered the minimal clinically important difference in periodontal therapy. Similarly, the additional 0.9 mm CAL gain in Group B further supports the clinical relevance of adjunctive PDT for severe periodontitis.

An imbalance of inflammatory factors and disruption of bone metabolism are important manifestations of severe periodontitis. IL-1 *β* can induce apoptosis in periodontal ligament cells, promote osteoclast activation, and accelerate alveolar bone resorption. TNF-α can exacerbate gingival inflammation and disrupt the integrity of periodontal tissues, whereas IL-6 can amplify the inflammatory response, promote inflammatory cell infiltration, and simultaneously regulate bone metabolism disorders. As a marker of systemic inflammatory response, elevated CRP levels reflect the severity of periodontal inflammation and systemic inflammation. ALP participates in bone resorption and formation; elevated levels indicate active bone metabolism and enhanced bone resorption (21). Hence, investigating well-tolerated and efficacious multimodal therapeutic strategies is essential for optimizing clinical outcomes and improving patient well-being (22, 23). The results of this study showed that 6 months after treatment, Group B demonstrated greater improvements in both gingival crevicular fluid inflammatory factor levels and bone metabolism markers compared to Group A. Photodynamic therapy utilizes the selective accumulation of pathogenic bacteria within the periodontal pocket by photosensitizers; under laser irradiation at a specific wavelength, it generates reactive oxygen species, which can kill residual subgingival pathogenic bacteria, reduce the release of toxic products such as endotoxins, and inhibit the inflammatory response at its source (24). Reactive oxygen species can oxidatively damage the cell membranes and nucleic acids of pathogenic bacteria, thereby inhibiting their proliferation and metabolism. This reduces the synthesis and secretion of inflammatory factors, such as IL-1β, TNF-α, and IL-6, while simultaneously lowering CRP levels, alleviating systemic inflammatory responses, and mitigating the damage caused by inflammatory cells to periodontal tissues (25). In addition, photodynamic therapy can regulate the immune system, suppress excessive inflammatory reactions, thereby inhibiting osteoclast activation, reducing ALP activity, decreasing bone resorption, reducing CTX release, alleviating collagen degradation, relieving alveolar bone resorption, downregulating RANKL expression, upregulating OPG levels, inhibiting osteoclast differentiation and maturation, enhancing bone protection, and promoting the recovery of bone metabolism homeostasis (26).

Patients with severe periodontitis experience symptoms such as loose teeth, gum bleeding, and pain while chewing, which can impair basic oral functions such as chewing and pronunciation. They may also experience negative emotions, such as anxiety and inferiority, due to issues with facial aesthetics and social inconvenience, severely reducing their daily quality of life related to oral health. The results of this study showed that after 6 months of treatment, the OHIP-14 score in group B was lower than that in group A, indicating that periodontal basic treatment combined with photodynamic therapy can improve patients’ oral-related quality of daily life. Photodynamic therapy can kill residual subgingival pathogenic bacteria after basic periodontal treatment, remove plaque biofilm, inhibit recurrence, effectively alleviate clinical symptoms such as gingival redness, active bleeding, and periodontal abscess, reduce local oral pain and discomfort, and improve patients’ physiological pain experience. Simultaneously, photodynamic therapy can effectively reduce periodontal tissue damage, decrease periodontal probing depth, reduce clinical attachment loss, alleviate symptoms of tooth looseness, restore normal chewing function, reduce dietary restrictions caused by chewing difficulties, and improve functional limitations and physiological disorders (27). The non-invasive characteristics of photodynamic therapy can also reduce trauma and pain during treatment, improve patient compliance, promote continuous improvement of periodontal health, alleviate periodontal symptoms, and restore oral function. It can effectively improve patients’ negative emotions, such as anxiety and inferiority, caused by oral problems, reduce social avoidance behavior, and improve psychological discomfort and social barrier scores, thereby reducing overall scores. This study also indicated that the incidence of complications in the two groups was similar, indicating that combination therapy was safe and did not significantly increase the risk of complications.

However, this study has some limitations. This was a single-center study, all participants were from our hospital, and the sample was relatively homogeneous. Although selection bias was reduced by propensity score matching, there may still be confounding factors that were not included. Future extensive multi-institutional studies are needed to confirm the broader applicability of our results.

In summary, the combination of basic periodontal treatment and photodynamic therapy demonstrates good efficacy in treating severe periodontitis. It can effectively improve patients’ periodontal conditions, reduce subgingival plaque and gingival inflammation, enhance oral bone metabolism, and improve oral-related quality of life while maintaining good safety.

## Data Availability

The original contributions presented in the study are included in the article/Supplementary Material, further inquiries can be directed to the corresponding author.
